# Primary ocular adnexal MALT lymphoma involving the upper eyelid in a young woman: a case report and literature review

**DOI:** 10.3389/fimmu.2026.1920364

**Published:** 2026-10-05

**Authors:** Qiming Zhang, Liying Gu, Jing Yuan, Rui Wang, Yujuan Xu, Qiong Liu, Hongbo Li, Kefeng Shen, Ting Chen

**Affiliations:** 1 Department of Ophthalmology, The Sixth Hospital of Wuhan, Affiliated Hospital of Jianghan University, Wuhan, China; 2 Department of Pathology, The Sixth Hospital of Wuhan, Affiliated Hospital of Jianghan University, Wuhan, China; 3 Department of Radiology, The Sixth Hospital of Wuhan, Affiliated Hospital of Jianghan University, Wuhan, China; 4 Department of Hematology, Tongji Hospital, Tongji Medical College, Huazhong University of Science and Technology, Wuhan, China

**Keywords:** case report, clinical manifestations, eyelid, histopathological features, molecular abnormalities, primary ocular adnexal MALT lymphoma, treatment and management

## Abstract

Primary ocular adnexal MALT lymphoma (POAML) is a rare indolent small B-cell lymphoma that primarily involves the orbit, conjunctiva, and lacrimal gland, with a high risk of early misdiagnosis. Herein, we report a rare case of POAML arising in the right upper eyelid of a 35-year-old woman, who presented with progressive eyelid swelling. The diagnosis was established by surgical excision, histopathological examination, and immunoglobulin gene rearrangement analysis. No systemic involvement was identified, and the disease was staged as Ann Arbor IE. The patient received local radiotherapy (28 Gy) and achieved complete remission, remaining disease-free during a 3.5-year follow-up. Literature review indicates that POAML predominantly affects middle-aged and elderly individuals, although younger age and female predominance have been reported in Asian populations. Clinical manifestations vary by site and include orbital mass, proptosis, globe displacement, and ocular motility restriction. Conjunctival lesions typically present as hyperemia, chemosis, or a characteristic “salmon-pink” appearance. Histologically, POAML is characterized by diffuse proliferation of centrocyte-like cells, follicular colonization, and lymphoepithelial lesions (LELs), with CD20 and CD79a positivity. Previous studies have identified recurrent molecular alterations in POAML, including changes affecting the BCR/NF-κB, epigenetic, and NOTCH signaling pathways; rearrangements of *MALT1*, *BIRC3*, *BCL10*, and *FOXP1*; and chromosomal abnormalities such as trisomy 3 and trisomy 18. Chronic antigenic stimulation associated with infection or autoimmune disease is considered a major predisposing factor. Treatment is stage-dependent, with surgery, radiotherapy, or observation for early-stage disease and immunochemotherapy for advanced disease. This case underscores that POAML should be considered in the differential diagnosis of progressive eyelid swelling, even in young women, as timely and accurate diagnosis and appropriate treatment can lead to a favorable prognosis.

## 1 Introduction

Primary ocular adnexal mucosa-associated lymphoid tissue lymphoma (POAML) constitutes the major subtype of primary ocular adnexal lymphoma (POAL). The most frequently involved sites are the orbit (>40%), conjunctiva (35%–40%), lacrimal gland (10%–15%), eyelids (5%–10%), and lacrimal sac (<5%) (1, 2). Bilateral ocular involvement is relatively uncommon, occurring in 15%–23% of cases (3). POAML is an indolent low-grade B-cell lymphoma that typically presents with periocular swelling, proptosis, palpable masses, and characteristic conjunctival “salmon-pink patch” lesions. Approximately 80%–90% of patients present with Ann Arbor stage IE disease, whereas B symptoms (e.g., fever, night sweats, and weight loss) (<5%), distant dissemination, and histologic transformation are infrequent.

POAML mainly affects middle-aged and elderly individuals, with most patients diagnosed between 50 and 70 years of age (2, 4). A relatively higher proportion of younger and female patients has been reported in Asian cohorts (3). Owing to its rarity and nonspecific early symptoms, POAML is often misdiagnosed or underdiagnosed. At present, watch-and-wait strategies, surgical excision, and local radiotherapy constitute the main treatment modalities for POAML, while a small proportion of patients with disseminated disease may require systemic chemotherapy and/or immunotherapy.

In recent years, the reported incidence has increased, possibly due to population aging, advances in diagnostic techniques, chronic antigenic stimulation by infectious agents (e.g., *Chlamydia psittaci*) and autoimmune disorders, as well as occupational and environmental exposures (5). We herein report a rare case of POAML involving the eyelid of a 35-year-old woman and review the clinical manifestations, pathological characteristics, molecular alterations, pathogenesis, therapeutic management, and prognostic outcomes of POAML. Based on this rare case and an updated narrative review of the literature, this study aims to provide a reliable reference for clinicians to improve early recognition, accurate diagnosis, and standardized management of POAML.

## 2 Case presentation

A 35-year-old Han Chinese female was admitted to our ophthalmology department in November 2022 due to a 3-month progressive swelling of the superolateral right upper eyelid. She was otherwise in good general health, with no evidence of systemic lymphadenopathy or hepatosplenomegaly. Her medical history was unremarkable, with no prior autoimmune disease or malignancy and no family history of hereditary disorders. Routine laboratory tests, including complete blood counts, liver and renal function, lactate dehydrogenase (LDH), autoimmune antibodies, hepatitis virus serology, and abdominal ultrasonography, were within normal limits. The autoimmune antibody panel included antinuclear antibody (ANA), anti-SSA/Ro, anti-SSB/La, anti-thyroglobulin antibody (TgAb), and anti-thyroid peroxidase antibody (TPOAb). Hepatitis serology included hepatitis B surface antigen (HBsAg), hepatitis B core antibody (HBcAb), and hepatitis C virus antibody (anti-HCV). All of the above results were negative. Testing for *Chlamydia psittaci* is not routinely available at our institution owing to limited resources; therefore, testing was not performed in this case. Ophthalmologic examination revealed a soft, non-tender palpable mass in the right upper eyelid. The left eye was unremarkable. Visual acuity, intraocular pressure, slit-lamp examination, fundus examination, and optical coherence tomography (OCT) findings were all within normal limits. A chronological clinical timeline summarizing all key time points in the patient’s clinical course is presented in **Figure 1**. Orbital magnetic resonance imaging (MRI) revealed a lesion involving the right upper eyelid, measuring 0.8 × 2.4 cm in maximum cross-section, demonstrating isointense signal on T1-weighted imaging (T1WI) and slightly hyperintense signal on T2WI relative to extraocular muscle, with homogeneous mild-to-moderate contrast enhancement (**Figure 2A**). The lesion was confined to the subcutaneous tissue of the superolateral aspect of the right upper eyelid, superficial to the tarsal plate, without extension into the orbital cavity or involvement of the lacrimal gland or other intraorbital structures. No bony destruction was identified.

**Figure 1:**
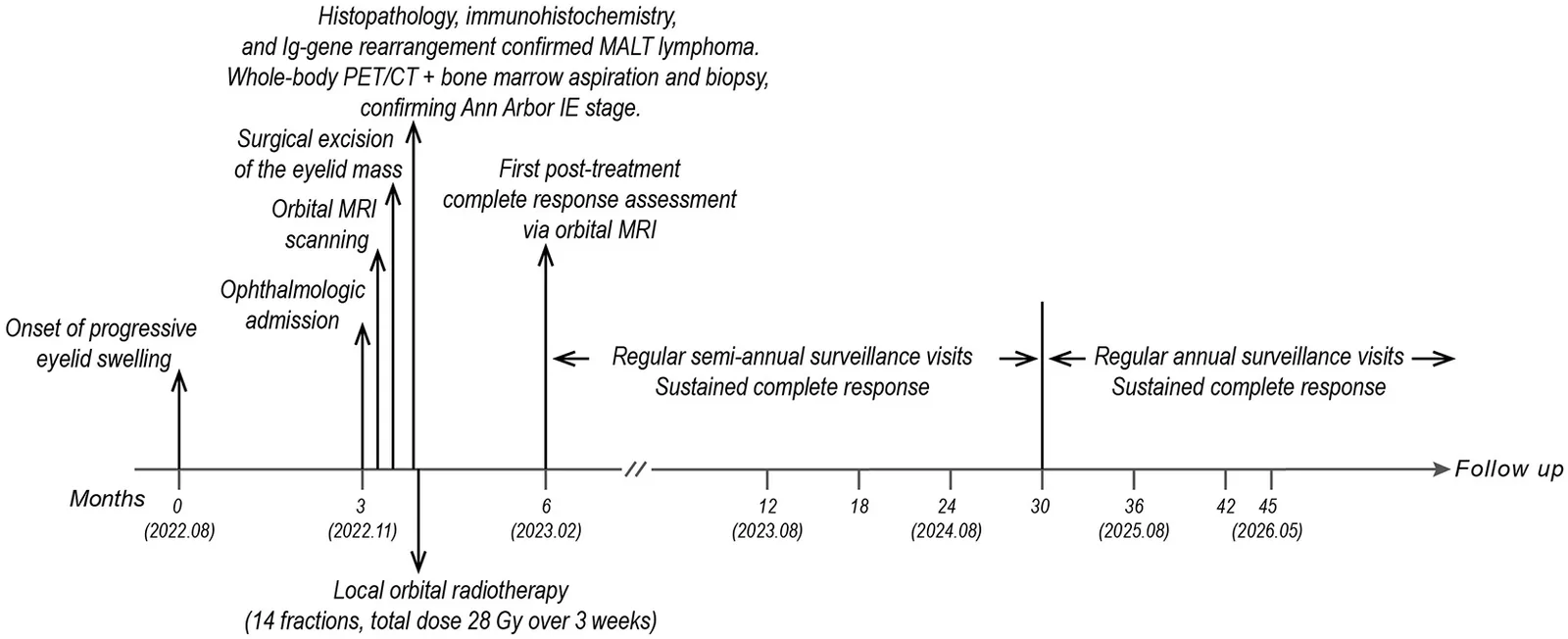
Chronological clinical timeline of the patient.

**Figure 2:**
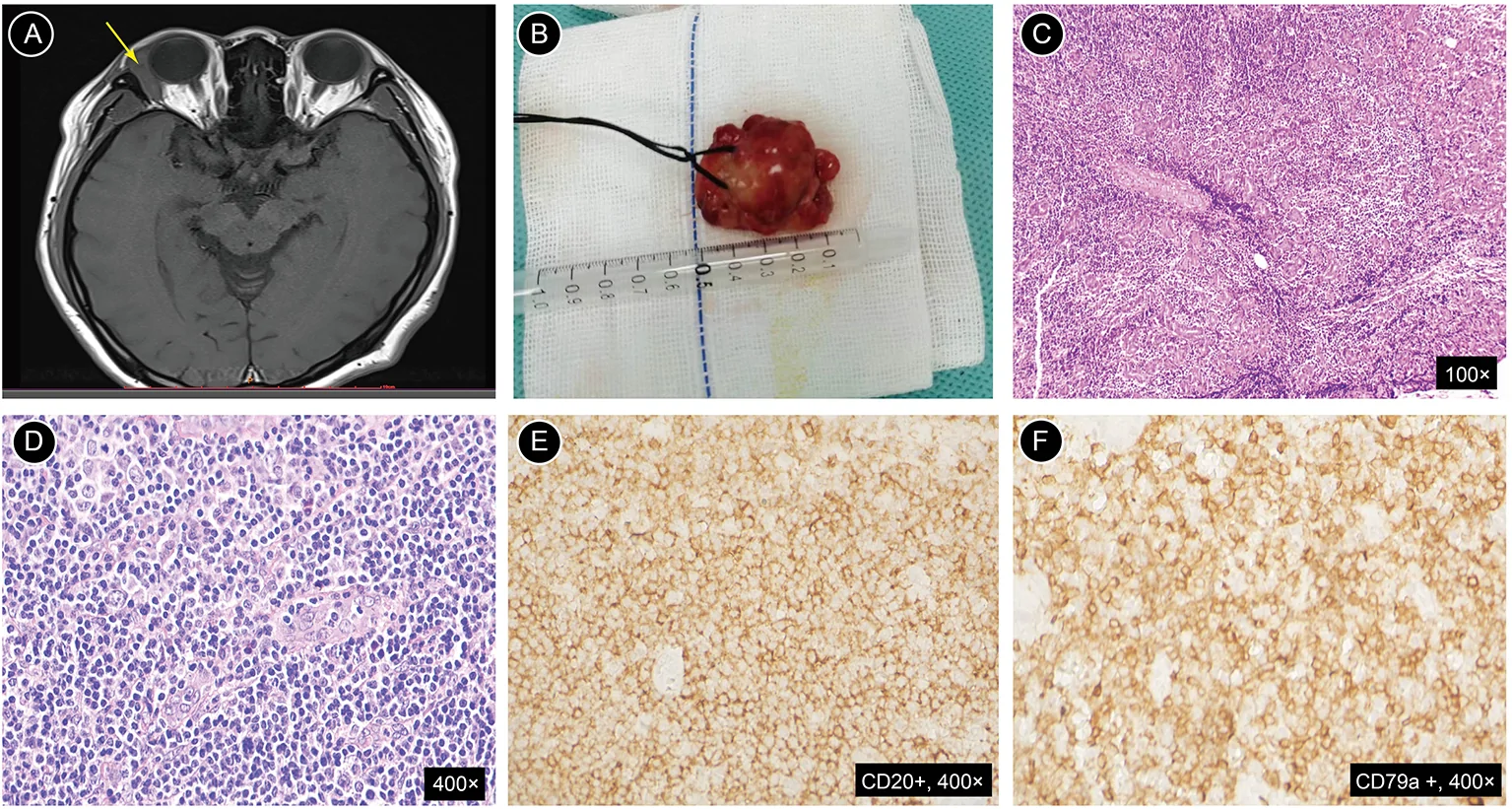
Clinical and pathological features of the patient. **(A)** Plain orbital MRI revealed an abnormal signal in the superolateral region of the right upper eyelid. The lesion appeared isointense on T1-weighted imaging (T1WI), with a maximum cross-sectional dimension of 0.8 × 2.4 cm. **(B)** Gross appearance of the surgically excised tumor specimen. **(C)** Histopathological examination revealed focal lymphoepithelial lesions (LELs) within the tumor tissue (H&E stain, ×100). **(D)** Diffuse infiltration of atypical small- to medium-sized lymphoid cells was observed (H&E stain, ×400). **(E, F)** Immunohistochemical staining showed strong positivity for CD20 and CD79a in the tumor cells (IHC, ×400).

The patient underwent a complete excisional biopsy because the lesion was small and easily accessible. The procedure was performed for diagnostic purposes rather than as a radical surgical treatment (**Figure 2B**). Histopathological examination (**Figures 2C, D**) revealed a diffuse infiltrate of small- to medium-sized centrocyte-like cells admixed with scattered monocytoid cells, small lymphocytes, and lymphoplasmacytoid cells. The neoplastic cells expanded from the marginal zones and infiltrated the surrounding tissue. Follicular colonization and focal lymphoepithelial lesions (LELs) were identified. Immunohistochemistry (**Figures 2E, F**) showed that the neoplastic cells were positive for CD20, CD79a, and Bcl-2, and negative for Cyclin D1, CD10, CD23, and Bcl-6. CD21 staining highlighted the follicular dendritic-cell network, while scattered background lymphocytes were positive for CD3 and CD5. The Ki-67 proliferation index was approximately 15%. Genomic DNA extracted from the biopsy specimen was analyzed using a commercial multiplex PCR kit complying with the validated EuroClonality/BIOMED-2 guidelines, which demonstrated clonal immunoglobulin gene rearrangements involving *IgH* and *Igκ*. Due to financial limitations and lack of access to fresh tissue samples, the patient did not undergo flow cytometric immunophenotyping, karyotype analysis, fluorescence *in situ* hybridization (FISH), and next-generation sequencing (NGS). Based on these findings, a diagnosis of extranodal marginal zone B-cell lymphoma (EMZL) of mucosa-associated lymphoid tissue (MALT) type involving the right upper eyelid was rendered.

The patient was referred to the Department of Oncology for further evaluation. Bone marrow aspiration and biopsy were negative for lymphoma involvement, and whole-body positron emission tomography/computed tomography (PET/CT) showed no evidence of extraorbital or systemic involvement. Orbital MRI delineated the local anatomical extent of the eyelid lesion. Morphological features, immunohistochemical profiles, and positive clonality testing collectively corroborated the diagnosis of POAML. Subsequent negative whole-body PET/CT and bone marrow biopsy findings confirmed Ann Arbor stage IE disease. The patient was further stratified as AJCC ocular adnexal TNM stage T3N0M0, with a MALT-IPI score of 0, corresponding to the low-risk group. Histological examination of the surgical specimen demonstrated focally positive resection margins with microscopic lymphoma infiltration, whereas postoperative imaging revealed no evidence of macroscopic residual disease. Given the risk of persistent microscopic residual lymphoma, she subsequently received adjuvant involved-site radiotherapy (ISRT) targeting the right upper eyelid surgical bed, with a total dose of 28 Gy delivered in 14 fractions over three weeks, to reduce the risk of local recurrence. ISRT was delivered using three-dimensional conformal radiation therapy (3D-CRT) with 6-MV photons. The clinical target volume encompassed the surgical bed with a 1-cm margin, with deliberate sparing of the retrobulbar tissues. Custom tungsten shielding was used to protect the lens. The maximum lens dose was <8 Gy, and the mean lacrimal gland dose was <10 Gy. Treatment was completed without interruption in late December 2022. The only acute toxicity was grade 1 periorbital erythema, which resolved spontaneously. No treatment-related complications such as dry eye, cataract or cosmetic impairment were noted. There were no patient-reported symptoms or deterioration in quality of life. The patient tolerated radiotherapy well, with no other clinically significant adverse events.

The first post-treatment response assessment was conducted two months after radiotherapy completion. Sustained complete remission was confirmed by combined comprehensive ophthalmic examination and orbital MRI. Subsequent surveillance included orbital MRI, laboratory tests and ophthalmological evaluations, performed every 6 months in the first two years and every 12 months afterwards. The patient underwent regular clinical surveillance. As of May 2026, she remained in sustained complete remission without evidence of disease recurrence, and no late radiation-related toxicities were observed during follow-up.

## 3 Patient perspective

The patient first noticed progressive swelling of her right upper eyelid three months before hospital admission, which gradually affected her appearance and caused mild psychological anxiety. She had no ocular discomfort such as pain, blurred vision, or elevated intraocular pressure, and maintained normal daily activities throughout the early disease stage. After a series of systematic ophthalmological and imaging examinations, she fully understood the necessity of surgical excision for lesion diagnosis and treatment and actively cooperated with the entire examination and treatment process. Following pathological confirmation of POAML, she accepted the clinical recommendation of adjuvant localized radiotherapy without hesitation. The patient tolerated surgical treatment and radiotherapy well with no obvious adverse reactions. During the regular follow-up from February 2023 to May 2026, she experienced no disease recurrence or ocular discomfort. She is currently in stable physical and mental condition, with completely restored daily life and full relief of initial anxiety.

## 4 Discussion

This case describes a rare primary upper-eyelid POAML occurring in a 35-year-old woman, presenting as a localized superficial eyelid lesion. The diagnosis was comprehensively established by morphological assessment, immunohistochemistry, and clonality testing. Systemic staging work-up ruled out distant disease. Following ISRT, the patient achieved durable remission with fully preserved ocular function.

Primary ocular lymphomas comprise a rare and heterogeneous group of lymphoid malignancies arising primarily within intraocular structures (e.g., the vitreoretinal compartment and uveal tract) or the ocular adnexa (e.g., the orbit, conjunctiva, lacrimal gland, and eyelids) (6). It is broadly classified into two main subtypes: primary intraocular lymphoma (PIOL) and POAL. Ocular involvement secondary to lymphomas originating from other anatomical sites, such as central nervous system lymphoma, is excluded. The predominant pathological subtype of PIOL is high-grade diffuse large B-cell lymphoma (DLBCL), frequently of the activated B-cell (ABC) or MCD subtype, and is characterized by an aggressive clinical course and poor prognosis (7). Primary vitreoretinal lymphoma (PVRL), the most common form of PIOL, typically involves the vitreous and retina and, rarely, the optic nerve, in the absence of cerebral parenchymal involvement at diagnosis (8). In contrast, primary uveal lymphoma is a rare PIOL subtype involving the choroid, iris, or ciliary body and is most often of EMZL of MALT type, characterized by an indolent course and favorable outcome (9).

POAL refers to lymphomas arising primarily in ocular adnexal tissues, including the orbit, conjunctiva, lacrimal gland, and eyelids. It accounts for approximately 1%–2% of all non-Hodgkin lymphomas (NHLs) and 5%–15% of extranodal lymphomas (10). The predominant histological subtype of POAL is EMZL of MALT type, commonly referred to as POAML, which accounts for approximately 55%–60% of all POAL cases and is even more prevalent in Asian populations (1, 2, 11). Other common histological subtypes include follicular lymphoma (FL), DLBCL, and mantle cell lymphoma (MCL), whereas T-cell lymphomas account for only 2%–3% of cases (2).

In the present case, the diagnosis of POAML required differential diagnosis from other ocular inflammatory lesions, reactive lymphoid lesions, and lymphomas (4, 5). Several entities that may mimic POAML should be considered. Chalazion is a lipogranulomatous lesion that lacks neoplastic B-cell infiltration, follicular colonization, LELs, and B-cell clonality, all of which were identified in our case. Reactive lymphoid hyperplasia typically shows preserved follicular architecture and a polyclonal B-cell population without clonal immunoglobulin gene rearrangement; by contrast, our case demonstrated follicular colonization, architectural disruption, and clonal *IgH*/*Igκ* rearrangements. Idiopathic orbital inflammation is characterized by a mixed inflammatory infiltrate, whereas IgG4-related ophthalmic disease typically shows storiform fibrosis and abundant IgG4-positive plasma cells; neither condition demonstrates neoplastic B-cell clonality or B-cell-derived LELs.

Among small B-cell lymphomas, FL was excluded based on the absence of CD10 and Bcl-6 expression in the neoplastic cells. MCL was excluded by the absence of CD5 and Cyclin D1 expression in the neoplastic B cells. Chronic lymphocytic leukemia/small lymphocytic lymphoma (CLL/SLL) typically shows co-expression of CD5 and CD23 in malignant B cells, which was not observed in our case. Lymphoplasmacytic lymphoma typically lacks the MALT-type features of follicular colonization and LELs. Taken together, the characteristic morphology (centrocyte-like cells, follicular colonization, and focal LELs), immunophenotype, low Ki-67 proliferation index (15%), and clonal immunoglobulin gene rearrangements supported the diagnosis of POAML.

Previously reported eyelid POAML cases have generally involved middle-aged to older patients, with a slight female predominance. In contrast, our patient was a 35-year-old woman with a localized superficial upper-eyelid lesion and Ann Arbor stage IE disease; similar to previously reported early-stage cases, she achieved durable complete remission with preserved ocular function after ISRT. While favorable outcomes have generally been reported for early-stage eyelid POAML, our case adds to the limited literature on POAML confined to the eyelid in younger adult women.

This study has several important limitations. First, as a single-case report, the generalizability of our findings is limited. Our observation of eyelid POAML in a young Asian woman cannot be broadly extrapolated to the wider POAML population. Second, given the nature of case-report evidence, we cannot establish causal relationships between potential predisposing factors (e.g., chronic antigenic stimulation, autoimmune background, or infectious triggers) and lymphoma pathogenesis in this patient. Third, comprehensive molecular profiling, such as NGS and cytogenetic analysis, was not performed, which limits our ability to characterize the full spectrum of somatic mutations, chromosomal abnormalities, and clonal evolution in this tumor. Finally, owing to the single-patient design, we cannot directly compare the efficacy, safety, or tolerability of alternative treatment strategies, such as watch-and-wait, rituximab monotherapy, antibiotic therapy, or local radiotherapy. Larger multicenter cohort studies are needed to overcome these limitations and further validate our observations.

To place our findings in a broader context and address the limitations inherent to a single-case report, we also provide an up-to-date review of the literature on the clinical features, pathological and molecular abnormalities, pathogenesis, treatment and management, and prognosis of POAML.

### 4.1 Clinical features

POAML predominantly affects middle-aged and elderly individuals, with most cases occurring between 50 and 70 years of age (median age, approximately 65 years) (4). Females are more commonly affected, with a male-to-female ratio of 1:1.5–2.0. However, in East Asian populations, the disease may present at a slightly younger age and exhibit a male predominance (3). As an indolent lymphoma, POAML is usually confined to the ocular adnexa, whereas extraocular involvement occurs only in a minority of cases.

The most characteristic signs and symptoms of POAML include an orbital mass, proptosis, globe displacement or restricted ocular motility, conjunctival hyperemia and chemosis, and the presence of a characteristic “salmon-pink” conjunctival patch (1, 4, 12). Patients may also present with acute inflammatory manifestations, such as epiphora, pain, or ocular irritation, as well as diplopia, decreased visual acuity, eyelid swelling, or ptosis. In the early stage, however, these manifestations are often subtle or atypical, and many patients are initially misdiagnosed with chronic conjunctivitis, inflammatory pseudotumor, or reactive lymphoid hyperplasia. The median diagnostic delay of POAML is approximately 9 months (range, 4–36 months) (3). Systemic B symptoms are rare (3.6%), and bone marrow involvement may be observed in 7.4% of cases, while elevated serum LDH (4.5%) and β2-microglobulin (11.6%) levels are infrequent; the median Ki-67 proliferation index is approximately 10% (3, 13, 14).

### 4.2 Pathological features and molecular abnormalities

Pathological features (4, 5, 15): (1) Morphology: The tumor is mainly composed of a heterogeneous population of small- to medium-sized B cells, including centrocyte-like cells, monocytoid cells, small lymphocyte-like cells, and lymphoplasmacytoid cells. Variable plasmacytic differentiation may be present, along with scattered immunoblasts or centroblast-like cells. The neoplastic cells show diffuse proliferation or a sheet-like growth pattern, expanding from the marginal zone of follicles. Follicular colonization can also be observed, and LELs may be present in some cases. (2) Immunophenotype: CD20+, CD79a+, PAX5+, Bcl-2+, CD5−, CD10−, CD23−, Cyclin D1−, Bcl-6−. Immunoglobulin light chain restriction (*Igκ*/*Igλ*) may be demonstrated. CD21 staining reveals residual or disrupted follicular dendritic cell (FDC) networks. A small number of scattered CD3+ T lymphocytes are present. The Ki-67 index is generally low (<20%–30%). Based on the immunophenotypic profile, MALT lymphoma can usually be distinguished from other small B-cell lymphomas, including MCL (CD5+, Cyclin D1+), FL (CD10+), and CLL/SLL (CD5+, CD23+).

Molecular abnormalities (16, 17): (1) Gene mutations: recurrent genetic alterations predominantly involve the B-cell receptor (BCR)/NF-κB signaling pathway, including *TNFAIP3* (35%–40%), *MYD88* (5%–10%), *TBL1XR1* (5%–10%), *CARD11* (2%–5%), *BCL10*, *BIRC3*, *CD79A/B*, and *TRAF2/3*; epigenetic regulators, such as *KMT2D* (15%–20%), *CREBBP* (5%–10%), and *TET2* (2%–5%); the NOTCH signaling pathway, including *NOTCH2* (5%–10%) and *NOTCH1* (2%–5%); and immune evasion-related genes, such as *TNFRSF14* (2%–5%) and *CD274* (5%–10%). (2) Gene rearrangements: recurrent gene rearrangements include t(14;18)(q32;q21)/*IGH*::*MALT1* (5%–10%), t(11;18)(q21;q21)/*BIRC3*::*MALT1* (5%–10%), t(1;14)(p22;q32)/*IGH*::*BCL10* (rare), and t(3;14)(p14.1;q32)/*IGH*::*FOXP1* (rare). (3) Karyotype abnormalities: trisomy 3 (35%–40%) and trisomy 18 (15%–20%). (4) Biased IGHV gene usage: particularly IGHV4-34 (18%), IGHV3-23 (14%), IGHV3-30 (12%), and IGHV3-7 (9%) (**Figure 3**).

**Figure 3:**
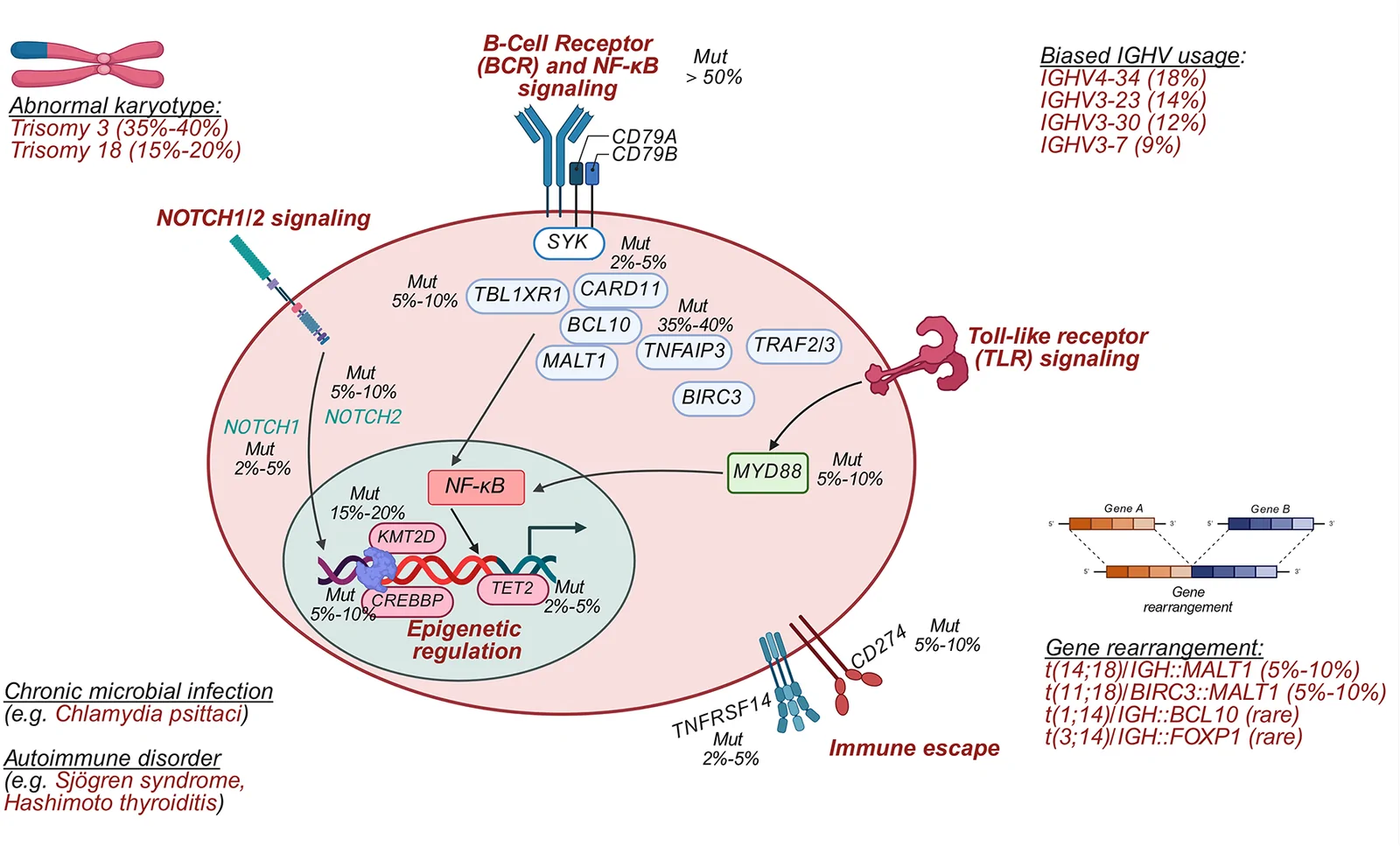
Etiological factors and molecular genetic features of POAML, including recurrent gene mutations, gene rearrangements, karyotypes, and biased IGHV gene usage. These molecular abnormalities are derived from previously published studies and were not assessed in the present patient.

### 4.3 Pathogenesis

POAML is an indolent low-grade B-cell lymphoma originating from marginal zone B cells. Its pathogenesis constitutes a multistep process driven by chronic antigenic stimulation and progressive genetic evolution (16). Generally, persistent microbial infection (e.g., *Chlamydia psittaci*) and/or autoimmune disorders (e.g., Sjögren’s syndrome and Hashimoto’s thyroiditis) establish a chronic inflammatory milieu that provides long-term antigenic stimulation within the ocular adnexal tissues, facilitates the acquisition of MALT, and induces dysregulated immune responses, ultimately leading to the emergence and expansion of autoreactive B cells (18). Over time, a fraction of these B cells accumulates cooperative oncogenic events, including chronic BCR stimulation, T-helper cell–mediated signals, and activation of other immune receptor pathways such as Toll-like receptor (TLR) signaling; as a result, these autoreactive B cells undergo clonal expansion and progressive evolution. Concurrently, the stepwise accumulation of somatic genetic abnormalities, including gene mutations, chromosomal rearrangements, cytogenetic alterations, and biased IGHV gene usage, further promotes lymphomagenesis. Together, these events confer resistance to apoptosis and enable malignant transformation. Through Darwinian-like clonal selection, these transformed B-cell clones gradually gain a survival advantage, progressively dominate the local microenvironment, and ultimately evolve into overt lymphoma (**Figure 4**).

**Figure 4:**
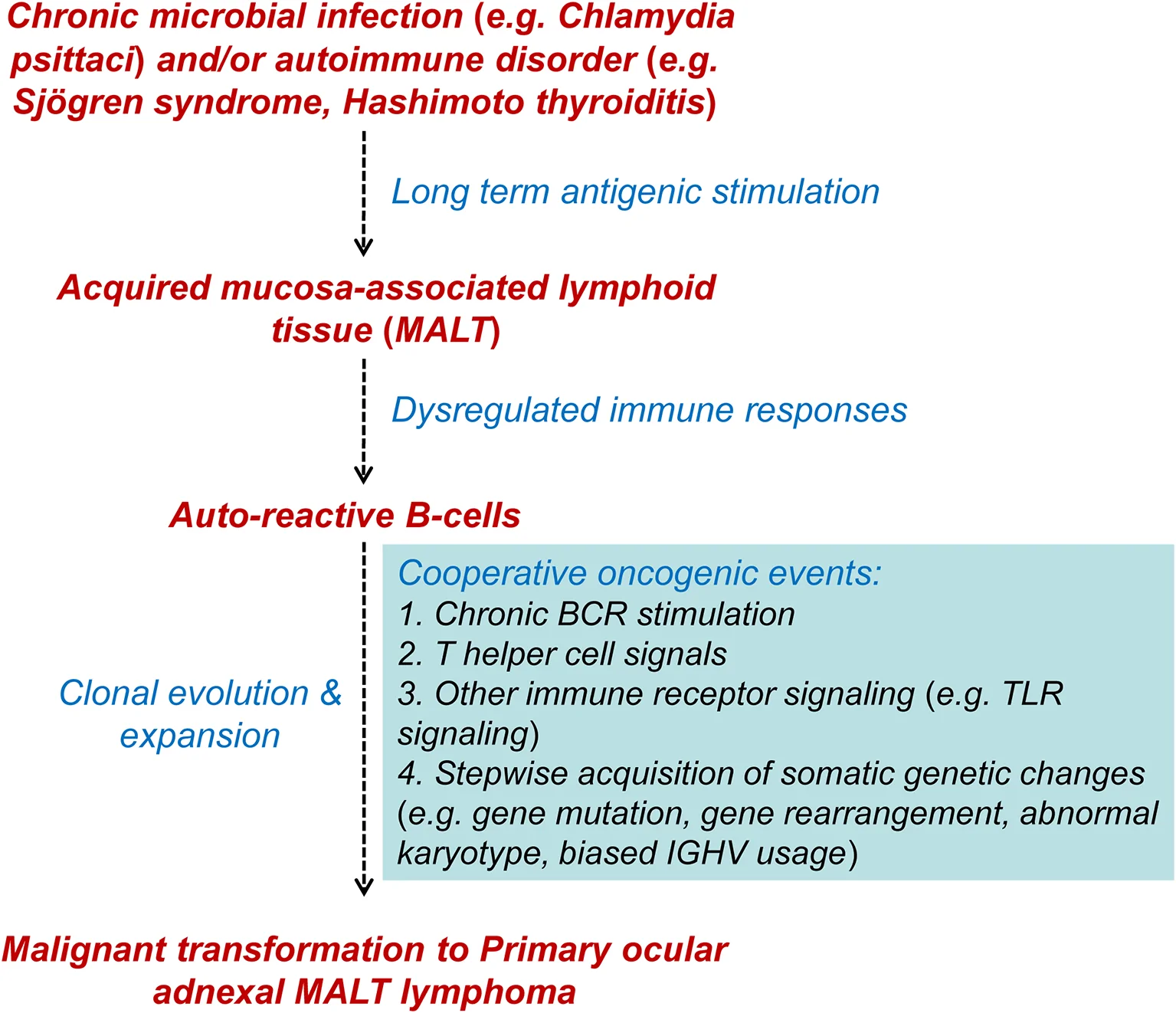
Proposed pathogenesis of POAML, involving chronic antigenic stimulation, cooperation of multiple genetic and environmental factors, and multistep malignant transformation.

### 4.4 Treatment, management, and prognostic outcomes

The treatment and management of POAML are primarily guided by clinical stage and risk stratification. Pretreatment evaluation typically includes comprehensive ophthalmologic examinations, laboratory and biochemical tests, bone marrow examination, and imaging studies, such as orbital MRI, CT of the neck, chest, abdomen and pelvis, or whole-body PET/CT. The two most commonly used staging systems are the Ann Arbor staging system and the American Joint Committee on Cancer (AJCC) tumor-node-metastasis (TNM) classification (19). Risk stratification is mainly based on prognostic models, including the International Prognostic Index (IPI) and the MALT Lymphoma International Prognostic Index (MALT-IPI) (20).

The primary goals of treatment are to preserve ocular function and cosmesis, reduce tumor burden, and control disease progression. The main therapeutic options include the following (3, 5, 12, 13): (1) For Ann Arbor stage I–II disease, ISRT (total dose: 24–30 Gy, fraction dose: 1.5–2.0 Gy) is considered the standard first-line treatment. Surgical intervention is mainly performed for diagnostic biopsy purposes, whereas radical excision is generally avoided to preserve ocular function and cosmetic appearance. Antibiotic therapy (e.g., doxycycline) may be indicated for *Chlamydia psittaci*-positive cases. However, the pathogen exhibits geographical heterogeneity in its distribution and is relatively uncommon in China. In selected patients with minimal residual disease, asymptomatic disease, or advanced age and frailty, a watch-and-wait strategy or rituximab monotherapy may also be appropriate. (2) For Ann Arbor stage III–IV disease, systemic therapy, including immunotherapy with or without chemotherapy, is recommended. Treatment options include rituximab monotherapy or rituximab-based chemoimmunotherapy regimens such as bendamustine-rituximab (BR), CHOP (cyclophosphamide, doxorubicin, vincristine, and prednisone), and CVP (cyclophosphamide, vincristine, and prednisone).

Response assessment is generally performed 3–6 months after completion of therapy. For patients achieving disease remission, follow-up evaluations are recommended every 3–6 months for the first two years and every 6–12 months thereafter. Overall, most patients with POAML have a favorable prognosis. In a large case series (1), the 5-, 10-, and 20-year disease-specific survival (DSS) rates were reported to be 96%, 91%, and 90%, respectively.

## 5 Conclusion

This case highlights that POAML should be considered in the differential diagnosis of persistent or progressive eyelid swelling, even in young patients with atypical clinical presentations. Definitive diagnosis requires careful histopathological and immunophenotypic evaluation, supplemented by genetic testing when necessary. In localized disease, timely and accurate diagnosis enables appropriate local treatment and may result in durable disease control. This case underscores the importance of maintaining clinical suspicion for POAML to avoid diagnostic delay and facilitate timely management.

## Data Availability

The original contributions presented in the study are included in the article/supplementary material. Further inquiries can be directed to the corresponding authors.

## References

[1] Hindsø TG Esmaeli B Holm F Mikkelsen LH Rasmussen PK Coupland SE . International multicentre retrospective cohort study of ocular adnexal marginal zone B-cell lymphoma. Br J Ophthalmol. (2020) 104:357–62. doi: 10.1136/bjophthalmol-2019-314008 31177189

[2] Vest SD Coupland SE Esmaeli B Finger PT Graue GF Grossniklaus HE . Specific location of ocular adnexal lymphoma and mortality: an international multicentre retrospective study. Br J Ophthalmol. (2023) 107:1231–8. doi: 10.1136/bjophthalmol-2021-320466 35512851

[3] Gao LR Li X Wang X Liang Y Wu Y Feng XL . Treatment and survival for patients with localized primary ocular adnexal extranodal marginal zone lymphoma. Leukemia. (2024) 38:914–7. doi: 10.1038/s41375-024-02227-5 38503872

[4] Kalogeropoulos D Papoudou-Bai A Kanavaros P Kalogeropoulos C . Ocular adnexal marginal zone lymphoma of mucosa-associated lymphoid tissue. Clin Exp Med. (2018) 18:151–63. doi: 10.1007/s10238-017-0474-1 28939925

[5] Sassone M Ponzoni M Ferreri AJ . Ocular adnexal marginal zone lymphoma: Clinical presentation, pathogenesis, diagnosis, prognosis, and treatment. Best Pract Res Clin Haematol. (2017) 30:118–30. doi: 10.1016/j.beha.2016.11.002 28288706

[6] Coupland SE . Molecular pathology of lymphoma. Eye (Lond). (2013) 27:180–9. doi: 10.1038/eye.2012.247 23222560 PMC3574248

[7] Soussain C Malaise D Cassoux N . Primary vitreoretinal lymphoma: a diagnostic and management challenge. Blood. (2021) 138:1519–34. doi: 10.1182/blood.2020008235 34036310

[8] Zhang X Peng X Liang D Chen Y Zhang W Zhang Y . Clinical guidelines for the diagnosis and treatment of vitreoretinal lymphoma in Chinese patients (2024). Eye Vis (Lond). (2025) 12:20. doi: 10.1186/s40662-025-00434-4 40414948 PMC12105337

[9] Aronow ME Portell CA Sweetenham JW Singh AD . Uveal lymphoma: clinical features, diagnostic studies, treatment selection, and outcomes. Ophthalmology. (2014) 121:334–41. doi: 10.1016/j.ophtha.2013.09.004 24144449

[10] Mulay K Honavar SG . An update on ocular adnexal lymphoma. Semin Diagn Pathol. (2016) 33:164–72. doi: 10.1053/j.semdp.2015.10.004 26972223

[11] Kirkegaard MK . Ocular adnexal lymphoma: Subtype-specific clinical and genetic features. Acta Ophthalmol. (2022) 100:3–37. doi: 10.1111/aos.15248 36196757

[12] Annibali O Sabatino F Mantelli F Olimpieri OM Bonini S Avvisati G . Review article: Mucosa-associated lymphoid tissue (MALT)-type lymphoma of ocular adnexa. Biology and treatment. Crit Rev Oncol Hematol. (2016) 100:37–45. doi: 10.1016/j.critrevonc.2016.01.009 26857986

[13] Desai A Joag MG Lekakis L Chapman JR Vega F Tibshirani R . Long-term course of patients with primary ocular adnexal MALT lymphoma: a large single-institution cohort study. Blood. (2017) 129:324–32. doi: 10.1182/blood-2016-05-714584 27789481

[14] Li X Wei J Zeng D Qi S Li B Guo W . Multicenter retrospective cohort study of 694 patients with ocular adnexal marginal zone B-cell lymphoma on behalf of the chinese ocular lymphoma collaborative group (COLCG). Blood. (2024) 144:4418. doi: 10.1182/blood-2024-206489

[15] Stefanovic A Lossos IS . Extranodal marginal zone lymphoma of the ocular adnexa. Blood. (2009) 114:501–10. doi: 10.1182/blood-2008-12-195453 19372259 PMC2713468

[16] Du MQ . EMZL at various sites: learning from each other. Blood. (2025) 145:2117–27. doi: 10.1182/blood.2024025794 39912633

[17] Laurent C Bertoni F . The biology of marginal zone lymphoma subtypes: challenge and relevance of classification. Blood. (2026) 147:105–14. doi: 10.1182/blood.2024028268 41026919

[18] Suarez F Lortholary O Hermine O Lecuit M . Infection-associated lymphomas derived from marginal zone B cells: a model of antigen-driven lymphoproliferation. Blood. (2006) 107:3034–44. doi: 10.1182/blood-2005-09-3679 16397126

[19] Coupland SE White VA Rootman J Damato B Finger PT . A TNM-based clinical staging system of ocular adnexal lymphomas. Arch Pathol Lab Med. (2009) 133:1262–7. doi: 10.5858/133.8.1262 19653722

[20] Thieblemont C Cascione L Conconi A Kiesewetter B Raderer M Gaidano G . A MALT lymphoma prognostic index. Blood. (2017) 130:1409–17. doi: 10.1182/blood-2017-03-771915 28720586

